# Reactive Nitrogen Species and Fibrinogen: Exploring the Effects of Nitration on Blood Clots

**DOI:** 10.3390/antiox14070825

**Published:** 2025-07-04

**Authors:** Francesca Nencini, Serena Borghi, Elvira Giurranna, Ilenia Barbaro, Niccolò Taddei, Claudia Fiorillo, Matteo Becatti

**Affiliations:** Department of Experimental and Clinical Biomedical Sciences “Mario Serio”, University of Firenze, Viale Morgagni 50, 50134 Firenze, Italy; francesca.nencini@unifi.it (F.N.); serena.borghi@unifi.it (S.B.); elvira.giurranna@unifi.it (E.G.); ilenia.barbaro@unifi.it (I.B.); niccolo.taddei@unifi.it (N.T.); claudia.fiorillo@unifi.it (C.F.)

**Keywords:** fibrin, fibrinogen, nitration, post-translational modifications, reactive nitrogen species, thrombosis

## Abstract

Reactive nitrogen species (RNS), particularly peroxynitrite (ONOO^−^), play a central role in post-translational modifications (PTMs) of proteins, including fibrinogen, a key component of the coagulation cascade. This review explores the structural and functional consequences of fibrinogen nitration, with a focus on its impact on clot formation, morphology, mechanical stability, and fibrinolysis. Nitration, primarily targeting tyrosine residues within functional domains of the Aα, Bβ, and γ chains, induces conformational changes, dityrosine crosslinking, and aggregation into high molecular weight species. These modifications result in altered fibrin polymerization, the formation of porous and disorganized clot networks, reduced mechanical resilience, and variable susceptibility to fibrinolysis. Moreover, nitrated fibrinogen may affect interactions with platelets and endothelial cells, although current evidence remains limited. Emerging clinical studies support its role as both a prothrombotic mediator and a potential biomarker of oxidative stress in cardiovascular and inflammatory diseases. Finally, we explore both pharmacological interventions, such as NOX inhibitors, and natural antioxidant strategies at counteracting fibrinogen nitration. Overall, fibrinogen nitration emerges as a critical molecular event linking oxidative stress to thrombotic risk.

## 1. Introduction

The reactive nitrogen species (RNS) encompass a diverse group of nitrogen-based oxidants that play critical roles in both physiological and pathological processes. Among the most studied RNS are nitric oxide (NO), peroxynitrite (ONOO^−^), and nitrogen dioxide (NO_2_•), which are known to mediate various cellular functions, including immune responses, vascular regulation, and oxidative stress [1,2,3]. While NO is often recognized for its vasodilatory and signaling properties, its interactions with superoxide anion (O_2_•−) can lead to the formation of ONOO^−^, a potent nitrating and oxidizing agent. This shift in reactive nitrogen balance has been implicated in numerous pathological conditions, including cardiovascular diseases, neurodegenerative disorders, and inflammatory responses [4,5,6,7,8,9,10,11].

Proteins, key molecular targets of RNS, can undergo various post-translational modifications (PTMs) such as nitration, oxidation, and S-nitrosylation [12,13,14]. Nitration involves the addition of a nitro (-NO_2_) group to amino acid residues, forming for example 3-nitrotyrosine [15,16]. These modifications can alter protein structure, function, and interaction with other biomolecules. Proteins subjected to nitration often exhibit impaired enzymatic activity, altered binding affinities, or enhanced susceptibility to degradation [17,18].

These effects are particularly significant in the context of blood coagulation, where fibrinogen, a key plasma glycoprotein, plays a central role in clot formation and stabilization [19,20,21].

Fibrinogen, a 340 kDa glycoprotein synthesized in the liver, is essential for hemostasis, providing the structural framework for blood clots. Under normal physiological conditions, thrombin-mediated cleavage of fibrinogen leads to the polymerization of fibrin monomers, which subsequently crosslink to form a stable clot [22,23]. However, exposure to RNS, particularly ONOO^−^, can result in the nitration of specific amino acid residues within the fibrinogen molecule. This modification may have profound effects on fibrin polymerization, clot stability, and susceptibility to fibrinolysis. Studies have suggested that nitrated fibrinogen forms fibrin networks with altered mechanical properties, potentially influencing thrombosis and clot resolution [24,25].

The implications of fibrinogen nitration extend beyond coagulation, as modified fibrinogen has been associated with pro-inflammatory responses and impaired wound healing [26,27]. Additionally, the presence of nitrated fibrinogen has been observed in pathological conditions such as atherosclerosis, ischemic stroke, chronic inflammatory diseases, and sepsis [28,29]. Understanding the biochemical and biophysical consequences of fibrinogen nitration is, therefore, crucial for elucidating its role in disease progression and identifying potential therapeutic targets.

This narrative review investigates the impact of RNS on fibrinogen, with particular emphasis on the mechanisms of nitration and the resulting functional effects on blood clot formation. To ensure a comprehensive and up-to-date synthesis, we conducted an extensive literature search across databases including PubMed, Scopus, and Web of Science. Preference was given to recent studies that introduced novel findings or perspectives not addressed in earlier reviews. Drawing on data from in vitro, in vivo, and clinical research, this review consolidates current knowledge on how nitrative modifications affect fibrinogen’s structure and function, with a focus on their implications for coagulation and fibrin clot dynamics.

By integrating biochemical, structural, and clinical insights, we aim to offer a thorough overview of the role of RNS-induced modifications in coagulation processes and their contribution to pathological conditions.

## 2. Reactive Nitrogen Species Overview

**Figure 1:**
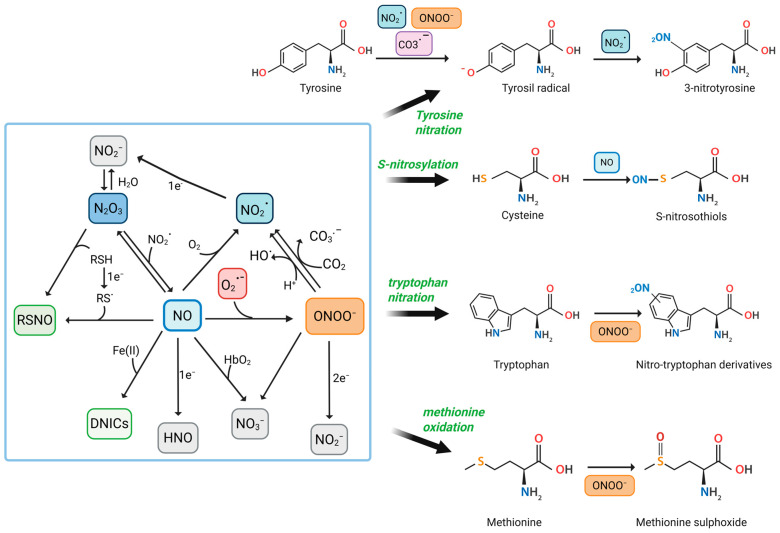
Reactive Nitrogen Species-Mediated Modifications of Amino Acids in Proteins. This figure illustrates the biochemical pathways by which RNS, such as NO, NO_2_•, and ONOO^−^, interact with specific amino acid residues to induce PTMs. Central to the diagram is the interplay between different RNS and their conversion pathways. CO_3_•^−^, carbonate anion radical; CO_2_, carbon dioxide; DNICs; Fe (II), iron (II); HbO_2_, oxyhemoglobin; HNO; NO_2_^−^; NO_3_^−^; N_2_O_3_; O_2_, molecular oxygen; O_2_•^−^; OH•, hydroxyl radicals; RSH, thiols; RS•, thiyl radical; RSNO, s-nitrosothiols. Created in BioRender. Nencini, F. (2025) https://BioRender.com/jn9qsfj, accessed on 20 June 2025.

RNS are a family of chemically reactive molecules derived from NO and nitrogen-containing compounds. They encompass both free radicals and non-radical oxidants and are involved in a variety of physiological and pathological processes. While NO and ONOO^−^ are the most well-studied, other significant RNS include NO_2_•, dinitrogen trioxide (N_2_O_3_), nitroxyl (HNO), nitrite (NO_2_^−^), nitrate (NO_3_^−^), and nitrosyl–metal complexes such as dinitrosyl iron complexes (DNICs) [30,31,32] (Figure 1).

NO is a small, lipophilic, and highly diffusible free radical produced enzymatically in mammalian cells by nitric oxide synthases (NOS). There are three main isoforms: neuronal NOS (nNOS), endothelial NOS (eNOS), and inducible NOS (iNOS). The first two are constitutively expressed and produced NO in small, regulated amounts in response to calcium–calmodulin signaling. In contrast, iNOS is induced under inflammatory conditions and produces much higher and sustained levels of NO [32,33,34]. NOS enzymes catalyze the conversion of L-arginine and O_2_ to L-citrulline and NO, requiring NADPH and several cofactors such as tetrahydrobiopterin (BH_4_), flavins (FAD, FMN), and heme [7,34].

Beyond enzymatic sources, NO can also be released from hemoglobin. Like oxygen, NO binds to the heme group in hemoglobin and is released under hypoxic conditions. Free NO then diffuses into vascular smooth muscle cells, promoting relaxation and vasodilation [35,36].

In the presence of oxygen, NO undergoes autoxidation to form NO_2_•, a strong oxidizing and nitrating agent [37,38]. Although this reaction is relatively slow under physiological conditions, it becomes significant in hydrophobic environments such as membranes, lipoproteins, and proteins, where concentrations of NO and oxygen are elevated [39].

Under physiological conditions, NO acts as a signaling molecule that modulates vasodilation, neurotransmission, platelet aggregation, and immune responses. Its primary signaling mechanism is through activation of soluble guanylate cyclase (sGC), leading to the production of cyclic GMP (cGMP) and downstream effects, including smooth muscle relaxation [33,40]. NO can also modify proteins via S-nitrosation and metal nitrosylation, contributing to post-translational regulation [36,41].

However, the biological impact of NO is highly context-dependent. In oxidative environments, particularly under conditions of inflammation or ischemia, NO reacts rapidly and in a diffusion-controlled manner with O_2_•^−^, forming ONOO^−^ [42]. This reaction occurs with an extremely high-rate constant (~10^10^ M^−1^s^−1^), making it kinetically favorable even at low concentrations of the reactants [43,44]. This coupling of reactive oxygen species (ROS) and NO metabolism constitutes a major source of RNS in cells. The formation of ONOO^−^ shifts the role of NO from a physiological messenger to a potential cytotoxic mediator [45,46].

ONOO^−^ is not a radical but a strong oxidant and nitrating agent. It exists in equilibrium with its protonated form, peroxynitrous acid (ONOOH), with a pKa of approximately 6.8 [32]. ONOO^−^ is highly unstable and can undergo several decomposition pathways. In aqueous solutions, it can isomerize to NO_3_^−^, but under biologically relevant conditions, it undergoes homolytic cleavage to form two highly reactive radicals: OH• and NO_2_•. These secondary species are capable of initiating lipid peroxidation, DNA strand breakage, and protein oxidation [47,48]. Alternatively, ONOO^−^ can react extremely rapidly with CO_2_, present in cells at millimolar concentrations. This reaction leads to the formation of nitrosoperoxycarbonate (ONOOCO_2_^−^), a transient intermediate that decomposes into NO_2_• and CO_3_•^−^, further amplifying its oxidative and nitrating potential [44,49].

ONOO^−^ can oxidize a broad range of biomolecular targets. It reacts with thiol groups on cysteine residues, forming sulfenic, sulfinic, or sulfonic acids, depending on the oxidative load. It can also nitrate tyrosine residues, generating 3-nitrotyrosine, a well-established biochemical footprint of nitrative stress [16,50]. This PTM can profoundly affect protein structure, function, and degradation. For instance, nitration of tyrosine in enzymes such as manganese superoxide dismutase (MnSOD) leads to enzymatic inactivation [16,43]. Nitrated fibrinogen has been shown to resist fibrinolysis and promote thrombosis, linking RNS to cardiovascular disease [28]. Additionally, ONOO^−^ interacts with transition metal centers in proteins (e.g., heme, iron–sulfur clusters), potentially disrupting electron transport and enzymatic catalysis [44,51].

Importantly, the biological outcome of ONOO^−^ formation is modulated by cellular compartmentalization and detoxification systems. For example, in the mitochondria, ONOO^−^ can impair respiratory chain complexes and induce mitochondrial permeability transition [32]. In macrophages, ONOO^−^ produced in the phagolysosome contributes to antimicrobial activity but may also cause tissue damage in chronic inflammation [44,52]. Enzymes such as peroxiredoxins, glutathione peroxidases, and thioredoxins serve as critical ONOO^−^ scavengers and regulators, limiting its harmful effects [36,53,54,55]. The interplay between NO, O_2_•−, and antioxidant defense systems determines whether RNS act as signaling molecules or mediators of oxidative injury [30,43].

Beyond NO and ONOO^−^, the following several additional RNS significantly contribute to redox biology:

- NO_2_• is a one-electron oxidant formed from NO autoxidation or ONOO^−^ decomposition. It is also found in environmental pollutants, including cigarette smoke [37,38]. NO_2_• can react with NO to form N_2_O_3_ [1] or it tends to form also dinitrogen tetroxide (N_2_O_4_), which is more soluble and rapidly breaks down into NO_2_^−^ and NO_3_^−^ [1]. Under physiological conditions, NO_2_• mainly reacts with biological molecules rather than forming N_2_O_4_, especially due to its high reactivity. In cells, NO_2_• acts as a strong one-electron oxidant, primarily targeting thiols, ascorbate, and urate. It can also trigger lipid peroxidation and modify fatty acids or proteins [30,56].
- N_2_O_3_ arises from the equilibrium between NO and NO_2_•, especially under low oxygen tension. N_2_O_3_ acts as a potent nitrosating agent, capable of converting thiols to RSNO, thus playing a central role in protein S-nitrosation and transnitrosation cascades [30].
- HNO, the one-electron reduced and protonated form of NO, possesses unique chemical properties distinct from NO. It can directly modify thiols, inactivate heme enzymes, and exert cardioprotective effects. Its role is emerging in redox signaling and pharmacology [31,57,58,59,60].
- NO_2_^−^ and NO_3_^−^ are often regarded as stable end-products of NO metabolism but are increasingly recognized as biologically active. In fasting individuals, plasma levels of NO_3_^−^ range from 20 to 40 μM, primarily originating from the reaction between NO and oxyhemoglobin, as well as from dietary sources [61]. In contrast, plasma NO_2_^−^ levels are much lower (typically 50–300 nM), due to its further conversion into NO, under hypoxic conditions, serving as a storage reservoir and participating in ischemic vasodilation. Moreover, NO_2_^−^ can act as a precursor for NO_2_• and N_2_O_3_ in acidic or peroxidase-rich environments [62,63].
- Nitrosyl–metal complexes, such as DNICs, are formed through the interaction of NO with transition metals and thiols. These complexes function as NO reservoirs and nitrosating intermediates and are implicated in protein regulation and cytotoxicity. DNICs are particularly abundant under inflammatory and nitrosative stress conditions and have been proposed to mediate long-range nitrosative signaling [64,65].
- RSNOs, although not classic RNS themselves, are crucial downstream products of RNS action. Formed via N_2_O_3_ or through metal-catalyzed NO transfer, they regulate redox-sensitive proteins via reversible S-nitrosation of cysteine residues. This modification modulates enzyme activity, protein–protein interactions, and subcellular localization [66,67].

Together, these species form a complex reactive nitrogen network, whose reactivity, lifespan, and diffusion are governed by local pH, redox status, availability of cofactors, and compartmentalization. The functional impact of RNS is therefore context-specific, ranging from precise signal modulation to pathological damage [3,68].

## 3. Protein Modifications Induced by RNS

RNS can modify various classes of biological macromolecules such as nucleic acids, lipids, and proteins. In nucleic acids, nitrative and oxidative attacks may lead to strand breaks and base modifications [69]. Lipid nitration induces changes in membrane fluidity and bioactive signaling lipid production [70]. However, proteins are the most frequent and consequential targets of RNS, undergoing a range of PTMs that significantly alter their structure and function [13,16,36].

Among the most studied RNS-induced modifications are the nitration of tyrosine residues, S-nitrosylation of cysteine thiols, and oxidative damage to methionine and tryptophan residues (Figure 1). These modifications can affect protein folding, stability, enzymatic activity, localization, and interactions, often tipping the balance from normal signaling to pathological outcomes.

### 3.1. Tyrosine Nitration

One of the most studied RNS-induced PTMs is the nitration of tyrosine residues to form 3-nitrotyrosine [15,43].

This occurs predominantly through the formation of ONOO^−^. The modification proceeds via a two-step free radical mechanism, as follows: first, the one-electron oxidation of the tyrosine phenolic ring generates a tyrosyl radical (Tyr•), followed by a radical–radical coupling with NO_2_•, forming 3-nitrotyrosine [15,17,71].

Also myeloperoxidase and eosinophil peroxidase catalyze tyrosine nitration using NO_2_^−^ and hydrogen peroxide under inflammatory conditions [72].

Tyrosine nitration alters the physicochemical properties of the residue, most notably decreasing its pKa and introducing steric and electrostatic changes, which can significantly affect protein conformation, stability, and function [73,74]. Functionally, this modification may impair or enhance enzymatic activity, as observed in mitochondrial proteins like cytochrome c and MnSOD, where nitration disrupts their redox properties [15]. Additionally, tyrosine nitration often occurs at phosphorylation sites, suggesting a competitive interaction that can interfere with key signaling pathways [71]. Nitrated proteins also tend to be more prone to proteolytic degradation or aggregation, processes linked to aging and degenerative diseases [15,43]. Moreover, nitrated tyrosine residues can serve as neoepitopes, potentially triggering immune responses and contributing to the development of autoimmune disorders [75,76,77,78,79]. The specificity of the tyrosine nitration is influenced by several factors, including the protein’s structure, local microenvironment, solvent accessibility of the residue, and proximity to metal catalytic centers [80]. Typically, only one or two tyrosines per protein are nitrated, even under high RNS stress [15,43].

3-nitrotyrosine is considered a reliable biomarker of nitrosative stress due to its chemical stability and detectability with various analytical techniques [79]. Elevated levels have been linked to cardiovascular, neurodegenerative, and inflammatory diseases [81,82]. However, despite the availability of several assays [83,84], current methods suffer from limited sensitivity and specificity, especially in complex biological samples. Significant variability in reported levels—often due to non-standardized techniques—hinders the definition of universal reference values. Standardized, validated protocols are therefore essential to improve measurement accuracy and ensure consistent, comparable results across studies.

### 3.2. S-Nitrosylation

S-nitrosylation is another significant PTM induced by RNS, in which a NO group is covalently attached to the thiol side chain of cysteine residues, forming S-nitrosothiols (SNOs) [36]. This reaction can occur directly via NO or indirectly through transnitrosylation reactions with S-nitrosylated proteins (such as thioredoxin or S-nitrosoglutathione, GSNO). Unlike nitration, S-nitrosylation is generally reversible and dynamically regulated by denitrosylases, including thioredoxin and GSNO reductase. It functions as a redox-sensitive regulatory switch, modulating key cellular processes such as apoptosis, metabolism, and immune responses [85,86,87].

Functionally, S-nitrosylation can influence enzymatic activity, protein–protein interactions, subcellular localization, and signal transduction pathways. For instance, S-nitrosylation of fatty acid synthase alters its dimerization and catalytic function [88], while modifications of neuronal proteins like N-methyl-D-aspartate (NMDA) receptor subunits and glyceraldehyde-3-phosphate dehydrogenase (GAPDH) affect synaptic plasticity and cell death mechanisms [89].

### 3.3. Other Modifications

Other aromatic and sulfur-containing amino acids, such as tryptophan and methionine, are vulnerable to PTMs induced by RNS.

Tryptophan, in particular, undergoes nitration and oxidation upon exposure to ONOO^−^, resulting in the formation of various nitrotryptophan isomers, including 4-, 5-, and 6-nitrotryptophan, with 6-nitrotryptophan being the predominant form [16,90,91]. These modifications can lead to protein inactivation, altered intrinsic fluorescence of tryptophan, changes in protein structure, and the generation of potential neo-epitopes that may trigger immune responses [90,92,93]. Nitration of tryptophan residues has been observed in mitochondrial proteins and could contribute to cellular dysfunction in pathological conditions such as aging and neurodegenerative diseases [94].

Similarly, methionine is prone to oxidation by RNS, particularly to methionine sulfoxide [95]. This modification is reversible and can significantly affect protein function, structure, and interactions [96]. The body has repair systems, such as methionine sulfoxide reductases (MsrA and MsrB), that can reduce methionine sulfoxide back to methionine, helping maintain redox homeostasis. However, in the presence of persistent oxidative stress, the accumulation of oxidized methionine can impair enzymatic functions, cellular signaling, and protein–protein interactions [97].

## 4. Consequences of Nitration on Fibrinogen Molecules and Clot Morphology

Although other abundant plasma proteins, particularly albumin and immunoglobulin G (IgG), are also susceptible to RNS attack, their reactivity is markedly lower than that of fibrinogen. Albumin is nitrated primarily at Cys34, with second-order rate constants four-to-six-fold lower than those reported for nitration of fibrinogen β-chain tyrosines [98,99]. Nitration of IgG seems to irreversibly alter the physicochemical properties of the molecule, potentially triggering immunological events such as inflammation and the induction of autoantibodies, as observed in various inflammatory disorders [100,101]. In contrast, fibrinogen possesses an unusually high tyrosine content (approximately 3.5% by mass), and many of these residues are solvent-exposed, features that make it a preferential target, or “nitrosative sink,” during conditions of oxidative stress [22,23,24].

Fibrinogen is a multidomain plasma glycoprotein composed of two sets of three polypeptide chains (Aα, Bβ, and γ) linked by 29 disulfide bonds, and plays a central role in coagulation by being converted into fibrin through thrombin-mediated cleavage of fibrinopeptides A and B. Its structure includes a central E domain and two distal D domains, connected via coiled-coil regions [20,21].

Exposure of fibrinogen to RNS leads to extensive PTMs that affect both its structure and function.

### 4.1. Fibrinogen Tyrosine Nitration: Insights from In Vitro and In Vivo Studies

One of the most prominent fibrinogen alterations is tyrosine nitration, observed in in vitro and in vivo studies (Table 1).

Several in vitro papers documented dose-dependent incorporation of nitro groups into tyrosine residues of fibrinogen [102,103,104,107,108,109,112,113,114]. In Nowak et al., spectrophotometric and fluorescence analyses have revealed that upon treatment with increasing concentrations of ONOO^−^ (10, 100, 1000 µM), approximately 0.5, 2, and 8 tyrosine residues per molecule are nitrated, respectively [105].

Also, exposure of fibrinogen to myeloperoxidase (MPO)/H_2_O_2_/NO_2_^−^, 3-morpholinosydnonimine (SIN-1), nitronium tetrafluoroborate (NO_2_BF_4_), and other nitric oxide donors (sodium nitroprusside (SNP) or ProliNONOate) resulted in tyrosine nitration growth [26,106,110,111].

Nitrated fibrinogen has been identified in various stress-related and pathological conditions. Ageing, for instance, is linked to increased production of nitroxidative agents and the accumulation of nitrated fibrinogen, among other modified proteins [119]. Using tandem mass spectrometry, Parastatidis et al. detected nitrated fibrinogen peptides in the plasma of smokers [116]. Similarly, Pignatelli et al. reported that cigarette smoking elevates oxidative stress, and during lung cancer development, RNS contribute to nitration and oxidation of plasma proteins [103].

Elevated levels of nitrated fibrinogen in plasma have been proposed as a potential biomarker for CAD. These increases are accompanied by changes in the physical properties of fibrinogen and fibrin, promoting a pro-thrombotic state [26]. Additionally, conditions such as acute respiratory distress syndrome [102], venous thromboembolism [28], and ischemic stroke [110,114] have also been associated with the presence of nitrated fibrinogen.

In contrast, the concentration of nitrated proteins in the plasma of multiple myeloma (MM) patients does not appear to differ significantly from that of healthy individuals [118]. However, fibrinogen nitration observed in experimental human endotoxemia has been shown to correlate with markers of inflammation and coagulation, suggesting it may act as a risk factor for cardiovascular complications [117]. Sepsis is an acute pathological state characterized by excessive ROS and RNS production, leading to endothelial dysfunction and disseminated intravascular coagulation. Elevated levels of 3-nitrotyrosine-modified proteins have been detected in the plasma of septic patients [29,120]. This supports a role for nitrative stress in promoting microvascular thrombosis in acute systemic inflammation, extending its relevance beyond chronic vascular disease.

### 4.2. Structural and Genetic Basis of Site-Selective Fibrinogen Nitration

Protein nitration depends largely on the structure of the protein and how accessible specific tyrosine residues are to RNS. Tyrosines buried within the protein’s core are generally protected, while those exposed to the surface or in flexible regions are more likely to be nitrated. This accessibility is often measured by solvent-accessible surface area, with higher values indicating greater susceptibility. Additionally, the local chemical environment, such as nearby charged residues or hydrogen bonds, can influence whether a tyrosine is reactive. Since proteins are dynamic, even normally hidden residues can become temporarily exposed. These structural factors make nitration a selective, non-random process that can significantly alter protein function [15,73,102,121,122].

Emerging evidence suggests that genetic variations within the fibrinogen gene cluster also influence susceptibility to nitration. The β-chain −455 G/A promoter polymorphism (rs1800790) is among the most studied; the A allele is linked to higher baseline fibrinogen levels and a 25% increase in circulating nitro-fibrinogen in smokers, indicating a gene–environment interaction driven by substrate availability [123,124,125]. The γ′ isoform, produced via alternative splicing of the FGG gene, includes a negatively charged C-terminal extension that partially shields tyrosine residues, making it less susceptible to nitration compared to the standard γA/γA isoform. While γ′ fibrinogen may offer protection under oxidative stress, it also alters clot properties, by slowing clot formation while increasing clot strength and resistance to fibrinolysis, potentially contributing to thrombosis in certain contexts. Thus, γ′ fibrinogen may play both protective and pathogenic roles depending on the oxidative and hemostatic environment [126,127,128,129,130,131]. Other polymorphisms, such as the FGB promoter variants (−148 C/T and −1420 G/A) and the BβArg448Lys, have been linked to changes in fibrinogen levels and clot architecture [132]. However, there is currently no direct evidence that these variants alter the susceptibility of fibrinogen tyrosines to nitration. Their influence is likely indirect—through increased substrate availability or structural modifications that may affect how fibrinogen responds to oxidative stress. Together, these genetic variants may serve as modulators of the impact of RNS on fibrinogen function, potentially contributing to individual differences in thrombotic risk and cardiovascular disease susceptibility.

### 4.3. Site-Specific Nitration of Fibrinogen Molecule

**Figure 2:**
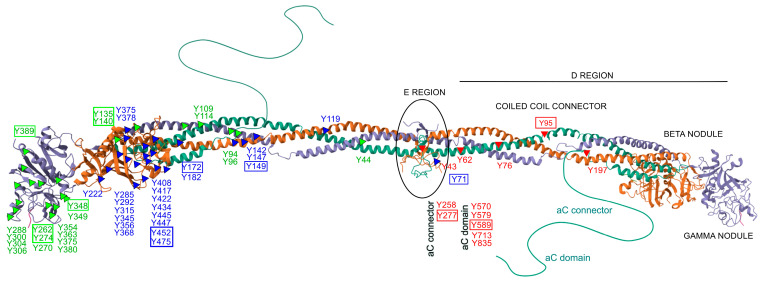
Structural distribution of nitrated tyrosine residues within the human fibrinogen molecule. The figure depicts the molecular structure of fibrinogen, composed of three pairs of polypeptide chains: Aα (FIBA, green), Bβ (FIBB, orange), and γ (FIBG, gray). Known tyrosine (Y) nitration sites are marked along each chain: red triangles indicate modified tyrosines on the Aα chain, blue triangles on the Bβ chain, green triangles on the γ chain. Tyrosine residues boxed in color denote sites consistently reported as nitrated in multiple independent in vitro and/or in vivo studies. Relevant structural regions are labeled, including the central E region, coiled-coil connectors, D regions with β and γ nodules, and the flexible αC domains. This representation highlights the positional clustering of nitration-susceptible residues in surface-exposed and functionally significant domains. The structure is based on the NMR model of fibrinogen (PDB ID: 3GHG).

Only a limited number of studies have specifically investigated site-specific nitrations [24,25,27,133]. Within the fibrinogen molecule (Figure 2), Tang et al. identified 26 nitrated tyrosine residues across the three chains in vitro, among 39 tyrosines observed. Importantly, nitration was not randomly distributed but preferentially occurred at surface-exposed tyrosines, particularly those located in flexible loop regions. These structural features are functionally relevant, as they are implicated in fibrin polymerization, thrombin interaction, and crosslinking by factor XIIIa, highlighting how molecular conformation strongly influences susceptibility to nitration—even under controlled in vitro conditions [134]. In a complementary study, Luo et al. demonstrated a concentration-dependent increase in nitration of the γ-chain following ONOO^−^ exposure, identifying five major modified residues. Four of these—Y262, Y274, Y348, and Y363—are located in the C-terminal region (domain 2), while Y96 resides in the N-terminal region. These nitrated residues were all situated in highly accessible loop structures, reinforcing their potential role in modulating fibrinogen’s physiological function [135]. Nowak et al. further showed that the Aα chain is particularly susceptible to both nitration and oxidation, with modifications affecting critical regions such as the αC domain and the D domain [105]. These regions are functionally significant for lateral aggregation of protofibrils, activation of factor XIII, cell adhesion, and for harboring binding sites for t-PA, plasminogen, α2-antiplasmin, and PAI-2—key regulators of fibrinolysis. In line with this, Meredith et al. observed dose-dependent increases in nitrotyrosine and methionine sulfoxide across all fibrinogen chains following treatment with SIN-1 [136].

Additional insights were provided by Parastatidis et al., who used affinity-based enrichment combined with high-resolution tandem mass spectrometry to identify βY292 and βY422 as frequently nitrated residues within the D domain of the Bβ chain. These modifications were not only detected in circulation but were also shown to be incorporated into thrombi in vivo. Using a murine model of carotid artery photochemical injury, the authors demonstrated that exogenously administered nitrated human fibrinogen localized within developing thrombi at sites of vascular injury. Immunofluorescence confirmed colocalization with platelet- and fibrin-rich areas, and the presence of nitrated fibrinogen was associated with increased clot density and reduced susceptibility to fibrinolysis [116].

In another study, Medeiros et al. identified 20 different 3-nitrotyrosine residues on fibrinogen nitrated in vitro, with nitration patterns influenced by the ONOO^−^ -to-fibrinogen molar ratio. Among nine tyrosine residues assessed in vivo in plasma samples, only βY452, βY475, and γY380 showed significantly elevated nitration levels in patients with ischemic stroke compared to individuals with non-inflammatory conditions. These results suggest that, under physiological conditions, nitration is selective and may preferentially affect residues involved in fibrin polymerization and clot stability, supporting their potential as biomarkers of thrombotic risk and as targets for therapeutic intervention [114].

Across studies, several tyrosine residues have emerged as consistently modified, including αY95, αY277, αY589, βY71, βY149, βY172, βY452, βY475, γY135, γY140, γY262, γY274, γY348, and γY389. These residues are distributed across essential structural domains of fibrinogen and carry distinct functional roles. For example, αY95 is located in the N-terminal E domain and is involved in thrombin-mediated cleavage and fibrinopeptide release, while αY277 and αY589 lie within the αC domain, crucial for fibrin fiber aggregation and crosslinking. Residues such as βY149, βY172, βY452, and βY475 are positioned within the D domain of the Bβ chain and contribute to protofibril formation and clot propagation. Similarly, γ-chain residues like γY262, γY274, γY348, and γY389 are located in the C-terminal γ-module and participate directly in knob–hole interactions essential for fibrin polymerization. Nitration at these functionally relevant sites may impair fibrin assembly, increase clot density, and reduce susceptibility to fibrinolysis, thereby promoting a prothrombotic phenotype. Overall, these findings underscore the importance of structural context in determining site-selective nitration and highlight the need for standardized approaches to assess its functional and diagnostic relevance.

### 4.4. Conformational Alterations and Clot Architecture Remodeling Induced by Nitration

Alongside tyrosine nitration, ONOO^−^ exposure leads to dityrosine crosslinking, which contributes to the formation of high molecular weight (HMW) aggregates. SDS-PAGE analysis revealed that exposing isolated human fibrinogen to ONOO^−^ induced oxidative modifications of the protein. Under reducing conditions, untreated control fibrinogen displayed the characteristic electrophoretic pattern with three distinct bands corresponding to the A (67 kDa), B (56 kDa), and γ (48 kDa) chains. In contrast, ONOO^−^-treated fibrinogen exhibited additional HMW bands (>120 kDa), indicating the formation of aggregates. These aggregates appeared above the typical A, B, and γ chains and were accompanied by a reduced intensity of these original bands, with the A chain being the most susceptible to modification. These aggregates arise through intermolecular bonding and appear in a concentration-dependent manner [105,107,108,112,113].

Spectrophotometric studies showed a fibrinogen marked hypochromicity at 280 nm following ONOO^−^ treatment, reflecting conformational rearrangements and diminished exposure of aromatic residues such as tyrosine and tryptophan [115]. Complementary intrinsic fluorescence measurements also reveal a decrease in emission intensity from these residues, suggesting disruption of tertiary structure and potential protein unfolding [109,110,112,115]. These alterations are further supported by bis-anilinonaphthalene sulfonate (ANS) binding assays, which indicate increased hydrophobic surface exposure, pointing to the exposure of internal hydrophobic domains and partial unfolding [115].

Carbonylation, a hallmark of oxidative stress, is another prominent modification resulting from ONOO^−^ exposure, particularly targeting lysine, arginine, proline, and threonine residues. Quantification through different assays consistently shows elevated carbonyl content in treated fibrinogen, correlating with irreversible oxidative damage and compromised function [105,106,111,115,118].

Only the study by Vadseth et al. reports data on RNS effects on the secondary structure of fibrinogen. The authors suggest a minimal impact on the secondary structure, showing preservation of α-helices and β-sheets [26].

Electron microscopy has further confirmed morphological alterations, revealing that nitrated fibrinogen displays irregular, aggregated ultrastructures with thick, twisted fibers and enlarged pores compared to the homogeneous fibrillar architecture of the native protein [26].

On the other hand, in Helms et al., fibrin clots formed in the presences of NO had a lower fiber density and larger fibrin fiber diameters. These changes were attributed to PTMs, such as S-nitrosation of thrombin and carbonylation of fibrinogen, which interfered with protofibril lateral aggregation. Interestingly, these modifications did not significantly affect the mechanical extensibility of the individual fibers [111].

The findings from Vadseth et al. showed that in vitro exposure of fibrinogen to nitrating agents led to fibrin networks composed of loosely organized, twisted bundles of thin fibers, with increased porosity and reduced stiffness (as indicated by a lower storage modulus G′). Clots were structurally weaker and more deformable, raising the possibility of increased susceptibility to mechanical disruption and microembolism formation [26].

In the study by Ill-Raga et al., structural analyses showed that nitration disrupted the typical elongated conformation of fibrinogen, inducing a shift toward globular, disordered aggregates. Increased clot stability was also observed, suggesting a possible contribution to ischemic injury [110].

Parastatidis et al. provided in vivo evidence that fibrinogen nitration alters clot structure. Specifically, high levels of nitration on fibrinogen of smokers were correlated with the appearance of clustered fiber architectures instead of uniform branching networks, and a significant increase in clot stiffness, as measured by viscoelastic parameters (G′ and G″). Notably, the diameter of individual fibrin fibers did not change significantly in their samples, suggesting that nitration alters clot morphology through changes in fiber organization rather than individual fiber dimensions [116].

Overall, nitration of fibrinogen leads to profound changes in its structural organization, including tyrosine nitration, dityrosine crosslinking, carbonylation, increased hydrophobicity, aggregation into HMW species, and altered fibrin architecture.

These modifications can lead to functional alterations with implications for prothrombotic and inflammatory states.

## 5. Functional Consequences of Fibrinogen Nitration

The post-translational nitration of fibrinogen induced by RNS has profound implications not only at the structural level, but also in terms of its biological function in coagulation and hemostasis.

### 5.1. Effects on Fibrinogen Polymerization and Fibrinolysis

Fibrinogen polymerization into fibrin is a finely regulated process initiated by thrombin. Thrombin cleaves fibrinopeptides A and B from the Aα and Bβ chains of fibrinogen, exposing specific binding sites within the central E domain. These sites interact with complementary “holes” located in the D domains of adjacent fibrinogen molecules, allowing their alignment into linear protofibrils. The subsequent removal of fibrinopeptide B facilitates interactions between the αC regions, promoting the lateral aggregation of protofibrils into thicker fibrin fibers. The clot is then stabilized by factor XIIIa, which covalently crosslinks γ- and α-chains, increasing its mechanical strength and resistance to fibrinolysis [22,23,137]. This polymerization process can be evaluated by measuring several kinetic and structural parameters. The rate of thrombin-catalyzed fibrin formation reflects the efficiency of conversion from fibrinogen to fibrin and determines the clotting time. The maximum velocity (Vmax) indicates the speed of lateral protofibril aggregation, while the lag phase reflects the time needed before fibril formation begins. The final turbidity or maximum absorbance (MaxAbs) of the clot provides insight into fibrin fiber thickness and overall clot density [138].

Fibrinogen nitration impacts polymerization kinetics in a biphasic, concentration-dependent manner. At low levels of nitration, induced by NO donors or low ONOO^−^ exposure, there is evidence of enhanced polymerization, reflected by a shortening of the lag phase and a steeper initial optical density slope. This may result from subtle conformational changes that increase the accessibility of binding sites in the E region or promote early alignment of fibrin monomers. However, at higher nitration levels, the process is markedly impaired. Key tyrosine residues become modified, leading to steric hindrance or loss of charge-based interactions required for proper D:E:D alignment. As a result, the lag phase is prolonged, and maximum turbidity is reduced, indicating delayed fiber formation and impaired lateral aggregation.

For instance, Nowak et al. reported that increasing concentrations of ONOO^−^ led to progressively more nitration and oxidation of fibrinogen, with associated reduction in clotting efficiency. At low levels of nitration (e.g., exposure to 10 μM ONOO^−^), clotting activity remained largely unaffected, but higher levels (≥100 μM) led to a marked decrease in polymerization efficiency [105].

The same results were observed in Ponczek et al. [106], which used NO_2_BF_4_ as the nitrating agent, and also in Bijak et al. [107,108].

Similarly, the in vitro study by Helms et al. showed that NO donor (ProliNONOate) exposure delayed fibrin clot formation by increasing the clotting time and reducing the initial polymerization rate [111]. In Ding et al., clotting time also increased with rising concentrations of manganese (Mn) (II), which was used in combination with ONOO^−^ to induce fibrinogen damage. The authors observed that higher levels of Mn (II) ions caused a greater degree of fibrinogen impairment [109].

Ill-Raga et al. extended these observations to in vivo human samples from ischemic stroke patients, showing that nitrotyrosinated fibrinogen formed clots that were slower to initiate formation, indicating enhanced clot stability at the expense of dynamic responsiveness [110].

In contrast, Vadseth et al. found that nitrated fibrinogen, both in vitro and in samples from patients with CAD, displayed a faster onset of clot formation but with altered fibrin architecture [26].

Also, in Gole et al., the exposure of fibrinogen to ONOO^−^ resulted in the acceleration of thrombin-catalyzed clot formation [102].

A direct correlation was observed between fibrinogen nitration and plasma clotting velocity in plasma samples collected before and 72 h after human endotoxin infusion, in an experimental model of endotoxemia. While the maximum turbidity remained unchanged, the rate of polymerization was higher following endotoxemia [117].

Nitration of fibrinogen accelerates fibrin clot formation even in smokers, where elevated levels of nitrated fibrinogen have been observed. This effect was reversed upon depletion of tyrosine-nitrated fibrinogen molecules [116].

Together, these results explain why nitrated fibrinogen forms clots that are mechanically abnormal—either prematurely dense or poorly structured—depending on the degree of modification, which may contribute to thrombotic or hemorrhagic complications in oxidative stress conditions.

The effect of nitration on fibrinolysis is complex and context dependent. In some studies, fibrin clots formed from nitrated fibrinogen show no significant change in susceptibility to plasmin-induced lysis, suggesting preservation of tissue plasminogen activator/plasminogen binding sites [26]. However, other findings indicate that nitration can retard fibrinolysis, potentially due to altered clot architecture and reduced permeability [110,116].

### 5.2. Effects on Platelet and Endothelial Cell Interactions

The interactions between nitrated fibrinogen and platelets or endothelial cells are complex and not yet fully understood.

Compared to the native form, fibrinogen treated with ONOO^−^ exhibits altered capacity to mediate platelet adhesion and aggregation. Both resting and ADP-activated platelets display reduced adhesion to ONOO^−^-modified fibrinogen, and the extent of ADP-induced platelet aggregation decreases in proportion to the degree of fibrinogen nitration [104,110].

However, Vadseth et al. reported no difference in the rate of ADP-induced platelet aggregation between control fibrinogen and fibrinogen exposed to SIN-1. Likewise, platelet adhesion to immobilized fibrinogen in multi-well plate assays was similar for both native and nitrated forms [26].

Regarding endothelial interactions, direct evidence on how nitrated fibrinogen affects endothelial cell function is limited. However, it is known that endothelial cells play a crucial role in regulating hemostasis and thrombosis, partly through the release of NO and prostacyclin, which inhibit platelet activation and aggregation [4,139]. The presence of nitrated proteins, including fibrinogen, could potentially influence these regulatory mechanisms, but further research is needed to elucidate these effects.

In summary, fibrinogen nitration exerts concentration-dependent effects on coagulation, fibrinolysis, and cellular interactions, highlighting its potential role in dysregulated hemostasis and thrombotic disease.

## 6. Targeting RNS Production

The pathological overproduction of RNS has prompted significant interest in strategies aimed at limiting their formation or neutralizing their effects.

### 6.1. NOX Inhibitors and Emerging Pharmacological Approaches

Among the most promising pharmacological targets are the NADPH oxidases (NOX), a family of membrane-bound enzyme complexes responsible for generating O_2_•−. Inhibiting NOX activity can thus indirectly reduce RNS burden by decreasing O_2_•− availability, limiting ONOO^−^ generation and downstream nitrative damage. Several NOX inhibitors, such as VAS2870, a pan-NOX inhibitor, and GKT137831 (Setanaxib), a dual NOX1/NOX4 inhibitor currently in phase-II trials, have shown beneficial effects in preclinical models by reducing oxidative damage and protein nitration and improving vascular and metabolic function [140,141,142]. However, the development of isoform-selective, potent, and safe NOX inhibitors remains a major focus in translational redox medicine.

Beyond NOX inhibitors, emerging strategies to reduce RNS-induced damage include also ONOO^−^ scavengers (e.g., Ebselen), Nrf2 activators (e.g., bardoxolone), and mitochondria-targeted antioxidants (e.g., MitoQ). These approaches aim to neutralize reactive species, enhance endogenous defenses, and limit oxidative injury at its source [143].

### 6.2. Natural Antioxidants Strategies

Natural antioxidant therapies serve to directly neutralize RNS or protect biomolecules from their effects. Endogenous systems are central to this defense, but exogenous antioxidants are increasingly explored for therapeutic use [144]. Although the effects of NOX inhibitors and other emerging strategies on fibrinogen nitration have yet to be clearly demonstrated, the ability of antioxidants to preserve fibrinogen integrity under conditions of nitrosative stress is well established.

Among the most studied dietary antioxidants is apigenin, a flavone found in various fruits, vegetables, and herbs. Apigenin has been shown to scavenge free radicals, inhibit ROS production, and enhance the activity of endogenous antioxidant enzymes [145,146]. The treatment of human fibrinogen with ONOO^−^ resulted in profound structural damage, loss of aromatic residue fluorescence, formation of carbonyl groups, and breakdown of subunit integrity. However, apigenin counteracted all these effects in a concentration-dependent manner [115]. Fluorescence studies showed that apigenin preserved tryptophan and tyrosine fluorescence intensity, suggesting protection against tertiary structure disruption. SDS-PAGE analysis demonstrated that apigenin also prevented the degradation of the Aα, Bβ, and γ chains, and reduced the formation of HMW aggregates [115].

Furthermore, apigenin significantly inhibited ONOO^−^-induced carbonylation, particularly for lysine, arginine, proline, and threonine residues, which are primary sites of irreversible oxidative damage. These protective effects were confirmed via 2,4-dinitrophenylhydrazine (DNPH)-based carbonyl quantification assays and bis-ANS-binding fluorimetry, which showed decreased surface hydrophobicity in apigenin-treated samples [115].

A similar protective profile is observed with (−)-epicatechin, a flavonoid abundant in green tea, dark chocolate, and red wine. It inhibits ONOO^−^-mediated tyrosine nitration far more effectively than it blocks oxidation reactions. In vitro, (−)-epicatechin reduces 3-nitrotyrosine formation in fibrinogen by up to 76% and attenuates dityrosine crosslinking. These effects are accompanied by partial restoration of fibrinogen’s clotting ability, as well as reductions in lipid peroxidation and protection of endothelial cells from oxidative injury [147,148,149,150].

Aronia melanocarpa (black chokeberry) berry extract, rich in anthocyanins and hydroxycinnamic acids, also exerts protective effects. Pre-incubation with this extract significantly inhibits HMW aggregate formation and fibrinogen tyrosine nitration, while restoring thrombin-induced fibrin polymerization. The efficacy of these actions is dose-dependent and linked to the extract’s high polyphenol content [108].

Further insights come from *Prunus spinosa* L. (blackthorn) flower extracts and their polyphenolic metabolites, which, even at low concentrations, prevent fibrinogen oxidation and nitration, limit aggregate formation, and maintain protein integrity [112].

In a similar vein, *Sorbus aucuparia* L. (rowanberry) fruit extracts demonstrate the ability to protect fibrinogen from oxidative damage, reduce thrombin enzymatic activity, and inhibit hyaluronidase in vitro. These effects are attributed to synergistic actions of flavonols, hydroxycinnamic acids, and proanthocyanidins, and reinforce the traditional use of rowanberries for cardiovascular support, especially in diabetic contexts [113].

Mechanistically, the protective activities of these extracts and isolated polyphenols were linked to multiple pathways, including direct scavenging of ONOO^−^ and its radical byproducts (NO_2_•, CO_3_•^−^), interference with tyrosyl radical formation, and reduction of protein carbonylation and surface hydrophobicity, thus preserving native protein folding and solubility. Importantly, none of the tested compounds or extracts exhibited pro-oxidant activity under experimental conditions.

Despite their antioxidant and antithrombotic effects, polyphenols suffer from poor oral bioavailability, due to limited absorption, rapid metabolism, and systemic elimination, which hampers their clinical impact on reducing fibrinogen nitration. Data on the long-term safety of high-dose supplementation remain sparse. To address this, various delivery strategies have been developed that significantly improve bioavailability and biological half-life. These include nanoformulations (such as liposomes, solid lipid nanoparticles, nanoemulsions, and nanocrystals), co-administration with absorption enhancers like piperine, encapsulation in biopolymers or protein carriers, and the design of prodrugs or synthetic analogs [151,152,153]. These approaches have shown promising results in improving solubility, stability, and systemic availability. Therefore, detailed pharmacokinetic profiling and dose–response studies are essential before considering their therapeutic application in humans. These data are critical to ensure efficacy and safety, and to define optimal dosing regimens for clinical use in preventing or mitigating RNS-mediated fibrinogen modification and thrombosis.

## 7. Conclusions

RNS, particularly NO and ONOO^−^, play a dual role in human physiology and pathology. While NO is a critical regulator of vascular tone, neurotransmission, and immune defense, its interaction with O_2_•− gives rise to ONOO^−^, a powerful oxidant and nitrating agent that can severely impair biomolecular integrity.

This review has outlined in detail the mechanisms of RNS formation, with a particular emphasis on NO and ONOO^−^, and described their molecular reactivity toward specific amino acid residues. Tyrosine and tryptophan nitration, S-nitrosylation, and methionine oxidation have been identified as major PTMs induced by RNS, each leading to distinct protein structural and functional consequences.

Among plasma proteins, fibrinogen nitration leads to a cascade of structural disruptions, including decreased aromatic residue exposure, enhanced carbonyl content, dityrosine crosslinking, and the formation of HMW aggregates. While its secondary structure may remain partially preserved, the tertiary and quaternary architecture is significantly altered, resulting in abnormal aggregation behavior.

Functionally, nitrated fibrinogen shows accelerated polymerization in in vivo studies, altered fibrin network architecture (with thinner, more porous, and less elastic fibers), and increased platelet adhesion. These changes collectively favor a pro-thrombotic state, which has been corroborated by observations of elevated nitrated fibrinogen levels in patients with cardiovascular and inflammatory conditions, as well as in acute conditions such as sepsis.

Importantly, we reviewed both natural antioxidant strategies, particularly polyphenols like apigenin and (−)-epicatechin, and pharmacological interventions aimed at mitigating fibrinogen nitration. While polyphenols offer promising protective effects through radical scavenging and inhibition of tyrosyl radical formation, their poor oral bioavailability limits their therapeutic impact, necessitating advanced formulation strategies such as nanoencapsulation or co-administration with absorption enhancers. In parallel, NOX inhibitors have emerged as targeted approaches capable of reducing superoxide production upstream, thereby attenuating ONOO^−^ formation at the source. Compounds targeting NOX2 and NOX4 isoforms in particular show promise in vascular and thrombotic pathologies linked to oxidative stress.

In conclusion, fibrinogen nitration represents a critical molecular link between nitro-oxidative stress and thrombotic disease. Its detection offers potential as a diagnostic and prognostic biomarker, while the modulation of this process, through both antioxidant and enzyme-targeted therapies, represents a viable avenue for clinical intervention. Future work should aim to translate these mechanistic insights into safe, effective treatments capable of attenuating RNS-mediated vascular damage.

## Figures and Tables

**Table 1:** Structural and functional effects of nitration on fibrinogen molecule.

Author	Method	PLT Aggregation and Binding	Fibrinogen Polymerization	Fibrinogen Nitration Markers	HMW Aggregates	Fibrinogen Structural Alterations	Fiber Characteristics	Fibrin Lysis
Gole et al. (2000) [102]	Fibrinogen + 1 mM ONOO^−^ + CO_2_		+	3nitrotyr				
Pignatelli et al. (2001) [103]	Fibrinogen + 1 mM ONOO^−^			3nitrotyr				
Nowak et al. (2002) [104]	Fibrinogen + 0.01, 0.1 and 1 mM ONOO^−^	-		3nitrotyr				
Vadseth et al. (2004) [26]	Fibrinogen + 50 mM SIN-1 + CO_2_	=	+	3nitrotyr dityr		CD	− Fiber diameter + Permeability − Density	=
	Fibrinogen 60 nM MPO +100 μM H_2_O_2_ +100 μM NO_2_^−^	=	+	3nitrotyr dityr		CD	− Fiber diameter + Permeability − Density	=
Nowak et al. (2007) [105]	Fibrinogen + 10 μM ONOO^−^ + CO_2_		=	3nitrotyr dityr PC	+			
	Fibrinogen + 100 or 1000 μM ONOO^−^ + CO_2_		-	3nitrotyr dityr PC	+			
Ponczek et al. (2008) [106]	Fibrinogen + 0.01 μM NO_2_BF_4_		+	3nitrotyr PC				
	Fibrinogen + 0.1 or 1 μM NO_2_BF_4_		-	3nitrotyr PC				
Bijak et al. (2012) [107]	Fibrinogen + 1 μM ONOO^−^		+	3nitrotyr	+			
	Fibrinogen + 10 μM or higher ONOO^−^		-	3nitrotyr	+			
Bijak et al. (2013) [108]	Fibrinogen + 100 μM ONOO^−^		-	3nitrotyr	+			
Ding et al. (2014) [109]	Fibrinogen + 8.7 μM ONOO^−^ + manganese		-	3nitrotyr		IF		
Ill-Raga et al. (2015) [110]	Fibrinogen + 100 μM SIN-1			3nitrotyr		IF		
	Fibrinogen + 10 mM SNP + 50 μM H_2_O_2_	-	-				+ stiffness	-
Helms et al. (2017) [111]	Fibrinogen + 5 μM NO donor ProliNONOate		-	3nitrotyr PC			+ Fiber diameter − Density	
Marchelak et al. (2021) [112]	Fibrinogen + 100 μM ONOO^−^			3nitrotyr	+	IF		
Rutkowska et al. (2021) [113]	Fibrinogen + 100 μM ONOO^−^			3nitrotyr	+			
Medeiros et al. (2021) [114]	Fibrinogen + 200, 100 and 10 mol of ONOO^−^ per mole of fibrinogen			3nitrotyr				
Farhana et al. (2024) [115]	Fibrinogen + 10 μM ONOO^−^			PC		IF UV spectrum		
Gole et al. (2000) [102]	Plasma from ARDS patients			3nitrotyr				
Pignatelli et al. (2001) [103]	Plasma from lung cancer patients and smokers			3nitrotyr				
Vadseth et al. (2004) [26]	Plasma from CAD patients			3nitrotyr				
	Fibrinogen from CAD patients		+	3nitrotyr				
Parastatidis et al. (2008) [116]	Fibrinogen from smokers <25 μmol nitrotyr/mol tyr		+	3nitrotyr			= Fiber diameter + stiffness	-
	Fibrinogen from smokers 25–50 μmol nitrotyr/mol tyr		+	3nitrotyr			− Density (Cluster of fibrin) = Fiber diameter + stiffness	-
	Fibrinogen from smokers >50 μmol nitrotyr/mol tyr		+	3nitrotyr			− Density (Cluster of fibrin) = Fiber diameter + stiffness	-
Heffron et al. (2009) [117]	Plasma from volunteers receiving 1 ng/kg LPS (endotoxemia)		+	3nitrotyr				
Martinez et al. (2012) [28]	Plasma from VTE patients			3nitrotyr				
Ill-Raga et al. (2015) [110]	Plasma from ischemic stroke patients			3nitrotyr		IF		
Nowak et al. (2017) [118]	Fibrinogen from MM patients			3nitrotyr PC				
Medeiros et al. (2021) [114]	Fibrinogen from ischemic stroke patients			3nitrotyr				

The table summarizes findings from multiple in vitro and in vivo studies analyzing the effects of nitrative PTMs on fibrinogen. “+” denotes an increase, “−” denotes a decrease, and “=“ denotes no change. ARDS, acute respiratory distress syndrome; CAD, coronary artery disease; CD, circular dichroism; dityr, dityrosine; IF, intrinsic fluorescence; MM, multiple myeloma; MPO, myeloperoxidase; PC, protein carbonyls; PLT, platelets; VTE, venous thromboembolism; 3nitrotyr, 3nitrotyrosine.

## Abbreviations

The following abbreviations are used in this manuscript:

CAD	Coronary Artery Disease
CD	Circular Dichroism
HMW	High Molecular Weight
IF	Intrinsic Fluorescence
LPS	Lipopolysaccharide
MM	Multiple Myeloma
Mn	Manganese
MPO	Myeloperoxidase
NO	Nitric Oxide
NOX	NADPH oxidase
NO2BF4	Nitronium Tetrafluoroborate
NOS	Nitric Oxide Synthase
PTMs	Post-Translational Modifications
RNS	Reactive Nitrogen Species
ROS	Reactive Oxygen Species
SIN-1	3-morpholinosydnonimine
SNP	Sodium Nitroprusside

## References

[1] Ford P.C., Miranda K.M. (2020). The solution chemistry of nitric oxide and other reactive nitrogen species. Nitric Oxide.

[2] Adams L., Franco M.C., Estevez A.G. (2015). Reactive nitrogen species in cellular signaling. Exp. Biol. Med..

[3] Di Meo S., Reed T.T., Venditti P., Victor V.M. (2016). Role of ROS and RNS Sources in Physiological and Pathological Conditions. Oxid. Med. Cell Longev..

[4] Jin R.C., Loscalzo J. (2010). Vascular Nitric Oxide: Formation and Function. J. Blood Med..

[5] Carlström M., Weitzberg E., Lundberg J.O. (2024). Nitric Oxide Signaling and Regulation in the Cardiovascular System: Recent Advances. Pharmacol. Rev..

[6] Levine A.B., Punihaole D., Levine T.B. (2012). Characterization of the role of nitric oxide and its clinical applications. Cardiology.

[7] Andrabi S.M., Sharma N.S., Karan A., Shahriar S.M.S., Cordon B., Ma B., Xie J. (2023). Nitric Oxide: Physiological Functions, Delivery, and Biomedical Applications. Adv. Sci..

[8] Dash U.C., Bhol N.K., Swain S.K., Samal R.R., Nayak P.K., Raina V., Panda S.K., Kerry R.G., Duttaroy A.K., Jena A.B. (2025). Oxidative stress and inflammation in the pathogenesis of neurological disorders: Mechanisms and implications. Acta Pharm. Sin. B.

[9] Tewari D., Sah A.N., Bawari S., Nabavi S.F., Dehpour A.R., Shirooie S., Braidy N., Fiebich B.L., Vacca R.A., Nabavi S.M. (2021). Role of Nitric Oxide in Neurodegeneration: Function, Regulation, and Inhibition. Curr. Neuropharmacol..

[10] Sharma J.N., Al-Omran A., Parvathy S.S. (2007). Role of nitric oxide in inflammatory diseases. Inflammopharmacology.

[11] Kim M.E., Lee J.S. (2025). Advances in the Regulation of Inflammatory Mediators in Nitric Oxide Synthase: Implications for Disease Modulation and Therapeutic Approaches. Int. J. Mol. Sci..

[12] Nakamura T., Oh C.K., Zhang X., Lipton S.A. (2021). Protein S-nitrosylation and oxidation contribute to protein misfolding in neurodegeneration. Free Radic. Biol. Med..

[13] Andrés C.M.C., Pérez de la Lastra J.M., Andrés Juan C., Plou F.J., Pérez-Lebeña E. (2022). Impact of Reactive Species on Amino Acids-Biological Relevance in Proteins and Induced Pathologies. Int. J. Mol. Sci..

[14] Suskiewicz M.J. (2024). The logic of protein post-translational modifications (PTMs): Chemistry, mechanisms and evolution of protein regulation through covalent attachments. Bioessays.

[15] Radi R. (2013). Protein tyrosine nitration: Biochemical mechanisms and structural basis of functional effects. Acc. Chem. Res..

[16] Jones L.H. (2012). Chemistry and biology of biomolecule nitration. Chem. Biol..

[17] Schopfer F.J., Baker P.R., Freeman B.A. (2003). NO-dependent protein nitration: A cell signaling event or an oxidative inflammatory response?. Trends Biochem. Sci..

[18] Lycus P., Einsle O., Zhang L. (2023). Structural biology of proteins involved in nitrogen cycling. Curr. Opin. Chem. Biol..

[19] Singh T., Hasan M., Gaule T.G., Ajjan R.A. (2025). Exploiting the Molecular Properties of Fibrinogen to Control Bleeding Following Vascular Injury. Int. J. Mol. Sci..

[20] Litvinov R.I., Weisel J.W. (2016). What Is the Biological and Clinical Relevance of Fibrin?. Semin. Thromb. Hemost..

[21] Litvinov R.I., Pieters M., de Lange-Loots Z., Weisel J.W. (2021). Fibrinogen and Fibrin. Subcell. Biochem..

[22] Kattula S., Byrnes J.R., Wolberg A.S. (2017). Fibrinogen and Fibrin in Hemostasis and Thrombosis. Arterioscler. Thromb. Vasc. Biol..

[23] Wolberg A.S. (2023). Fibrinogen and fibrin: Synthesis, structure, and function in health and disease. J. Thromb. Haemost..

[24] Nencini F., Bettiol A., Argento F.R., Borghi S., Giurranna E., Emmi G., Prisco D., Taddei N., Fiorillo C., Becatti M. (2024). Post-translational modifications of fibrinogen: Implications for clotting, fibrin structure and degradation. Mol. Biomed..

[25] de Vries J.J., Snoek C.J.M., Rijken D.C., de Maat M.P.M. (2020). Effects of Post-Translational Modifications of Fibrinogen on Clot Formation, Clot Structure, and Fibrinolysis: A Systematic Review. Arterioscler. Thromb. Vasc. Biol..

[26] Vadseth C., Souza J.M., Thomson L., Seagraves A., Nagaswami C., Scheiner T., Torbet J., Vilaire G., Bennett J.S., Murciano J.C. (2004). Pro-thrombotic state induced by post-translational modification of fibrinogen by reactive nitrogen species. J. Biol. Chem..

[27] Martinez M., Weisel J.W., Ischiropoulos H. (2013). Functional impact of oxidative posttranslational modifications on fibrinogen and fibrin clots. Free Radic. Biol. Med..

[28] Martinez M., Cuker A., Mills A., Lightfoot R., Fan Y., Tang W.H., Hazen S.L., Ischiropoulos H. (2012). Nitrated fibrinogen is a biomarker of oxidative stress in venous thromboembolism. Free Radic. Biol. Med..

[29] Bertozzi G., Ferrara M., Di Fazio A., Maiese A., Delogu G., Di Fazio N., Tortorella V., La Russa R., Fineschi V. (2024). Oxidative Stress in Sepsis: A Focus on Cardiac Pathology. Int. J. Mol. Sci..

[30] Möller M.N., Rios N., Trujillo M., Radi R., Denicola A., Alvarez B. (2019). Detection and quantification of nitric oxide-derived oxidants in biological systems. J. Biol. Chem..

[31] Pacher P., Beckman J.S., Liaudet L. (2007). Nitric oxide and peroxynitrite in health and disease. Physiol. Rev..

[32] Bartesaghi S., Radi R. (2018). Fundamentals on the biochemistry of peroxynitrite and protein tyrosine nitration. Redox Biol..

[33] Mazuryk O., Gurgul I., Oszajca M., Polaczek J., Kieca K., Bieszczad-Żak E., Martyka T., Stochel G. (2024). Nitric Oxide Signaling and Sensing in Age-Related Diseases. Antioxidants.

[34] Förstermann U., Sessa W.C. (2012). Nitric oxide synthases: Regulation and function. Eur. Heart J..

[35] Premont R.T., Reynolds J.D., Zhang R., Stamler J.S. (2020). Role of Nitric Oxide Carried by Hemoglobin in Cardiovascular Physiology: Developments on a Three-Gas Respiratory Cycle. Circ. Res..

[36] Yoon S., Eom G.H., Kang G. (2021). Nitrosative Stress and Human Disease: Therapeutic Potential of Denitrosylation. Int. J. Mol. Sci..

[37] Augusto O., Bonini M.G., Amanso A.M., Linares E., Santos C.C., De Menezes S.L. (2002). Nitrogen dioxide and carbonate radical anion: Two emerging radicals in biology. Free Radic. Biol. Med..

[38] Squadrito G.L., Postlethwait E.M. (2009). On the hydrophobicity of nitrogen dioxide: Could there be a "lens" effect for NO(2) reaction kinetics?. Nitric Oxide.

[39] Thomas D.D. (2015). Breathing new life into nitric oxide signaling: A brief overview of the interplay between oxygen and nitric oxide. Redox Biol..

[40] Nossaman B., Pankey E., Kadowitz P. (2012). Stimulators and activators of soluble guanylate cyclase: Review and potential therapeutic indications. Crit. Care Res. Pract..

[41] Wolhuter K., Whitwell H.J., Switzer C.H., Burgoyne J.R., Timms J.F., Eaton P. (2018). Evidence against Stable Protein S-Nitrosylation as a Widespread Mechanism of Post-translational Regulation. Mol. Cell.

[42] Thomas D.D., Ridnour L.A., Isenberg J.S., Flores-Santana W., Switzer C.H., Donzelli S., Hussain P., Vecoli C., Paolocci N., Ambs S. (2008). The chemical biology of nitric oxide: Implications in cellular signaling. Free Radic. Biol. Med..

[43] Ferrer-Sueta G., Campolo N., Trujillo M., Bartesaghi S., Carballal S., Romero N., Alvarez B., Radi R. (2018). Biochemistry of Peroxynitrite and Protein Tyrosine Nitration. Chem. Rev..

[44] Prolo C., Piacenza L., Radi R. (2024). Peroxynitrite: A multifaceted oxidizing and nitrating metabolite. Curr. Opin. Chem. Biol..

[45] Szabó C., Ischiropoulos H., Radi R. (2007). Peroxynitrite: Biochemistry, pathophysiology and development of therapeutics. Nat. Rev. Drug Discov..

[46] Ferrer-Sueta G., Radi R. (2009). Chemical biology of peroxynitrite: Kinetics, diffusion, and radicals. ACS Chem. Biol..

[47] Goldstein S., Merényi G. (2008). The chemistry of peroxynitrite: Implications for biological activity. Methods Enzymol..

[48] Ferdinandy P. (2006). Peroxynitrite: Just an oxidative/nitrosative stressor or a physiological regulator as well?. Br. J. Pharmacol..

[49] Pavlovic R., Santaniello E. (2007). Peroxynitrite and nitrosoperoxycarbonate, a tightly connected oxidizing-nitrating couple in the reactive nitrogen-oxygen species family: New perspectives for protection from radical-promoted injury by flavonoids. J. Pharm. Pharmacol..

[50] Ducrocq C., Blanchard B., Pignatelli B., Ohshima H. (1999). Peroxynitrite: An endogenous oxidizing and nitrating agent. Cell Mol. Life Sci..

[51] Radi R. (2013). Peroxynitrite, a stealthy biological oxidant. J. Biol. Chem..

[52] Prolo C., Alvarez M.N., Radi R. (2014). Peroxynitrite, a potent macrophage-derived oxidizing cytotoxin to combat invading pathogens. Biofactors.

[53] Perkins A., Nelson K.J., Parsonage D., Poole L.B., Karplus P.A. (2015). Peroxiredoxins: Guardians against oxidative stress and modulators of peroxide signaling. Trends Biochem. Sci..

[54] Sharapov M.G., Gudkov S.V., Lankin V.Z., Novoselov V.I. (2021). Role of Glutathione Peroxidases and Peroxiredoxins in Free Radical-Induced Pathologies. Biochemistry.

[55] Arnér E.S., Holmgren A. (2000). Physiological functions of thioredoxin and thioredoxin reductase. Eur. J. Biochem..

[56] Ford E., Hughes M.N., Wardman P. (2002). Kinetics of the reactions of nitrogen dioxide with glutathione, cysteine, and uric acid at physiological pH. Free Radic. Biol. Med..

[57] Bartberger M.D., Liu W., Ford E., Miranda K.M., Switzer C., Fukuto J.M., Farmer P.J., Wink D.A., Houk K.N. (2002). The reduction potential of nitric oxide (NO) and its importance to NO biochemistry. Proc. Natl. Acad. Sci. USA.

[58] Smulik R., Dębski D., Zielonka J., Michałowski B., Adamus J., Marcinek A., Kalyanaraman B., Sikora A. (2014). Nitroxyl (HNO) reacts with molecular oxygen and forms peroxynitrite at physiological pH. Biological Implications. J. Biol. Chem..

[59] Bianco C.L., Toscano J.P., Bartberger M.D., Fukuto J.M. (2017). The chemical biology of HNO signaling. Arch. Biochem. Biophys..

[60] Fukuto J.M., Switzer C.H., Miranda K.M., Wink D.A. (2005). Nitroxyl (HNO): Chemistry, biochemistry, and pharmacology. Annu. Rev. Pharmacol. Toxicol..

[61] Bescos R., Gallardo-Alfaro L., Ashor A., Rizzolo-Brime L., Siervo M., Casas-Agustench P. (2025). Nitrate and nitrite bioavailability in plasma and saliva: Their association with blood pressure—A systematic review and meta-analysis. Free Radic. Biol. Med..

[62] DeMartino A.W., Kim-Shapiro D.B., Patel R.P., Gladwin M.T. (2019). Nitrite and nitrate chemical biology and signalling. Br. J. Pharmacol..

[63] Kim-Shapiro D.B., Gladwin M.T. (2014). Mechanisms of nitrite bioactivation. Nitric Oxide.

[64] Holloway L.R., Li L. (2013). The Preparation, Structural Characteristics, and Physical Chemical Properties of Metal-Nitrosyl Complexes. Struct. Bond..

[65] Vanin A.F. (2021). Physico-Chemistry of Dinitrosyl Iron Complexes as a Determinant of Their Biological Activity. Int. J. Mol. Sci..

[66] Upchurch G.R., Welch G.N., Loscalzo J. (1995). S-nitrosothiols: Chemistry, biochemistry, and biological actions. Adv. Pharmacol..

[67] Broniowska K.A., Hogg N. (2012). The chemical biology of S-nitrosothiols. Antioxid. Redox Signal.

[68] Thomson L. (2015). 3-nitrotyrosine modified proteins in atherosclerosis. Dis. Markers.

[69] Andrés C.M.C., Lastra J.M.P., Juan C.A., Plou F.J., Pérez-Lebeña E. (2023). Chemical Insights into Oxidative and Nitrative Modifications of DNA. Int. J. Mol. Sci..

[70] Oliveira M.C., Yusupov M., Bogaerts A., Cordeiro R.M. (2020). How do nitrated lipids affect the properties of phospholipid membranes?. Arch. Biochem. Biophys..

[71] Griswold-Prenner I., Kashyap A.K., Mazhar S., Hall Z.W., Fazelinia H., Ischiropoulos H. (2023). Unveiling the human nitroproteome: Protein tyrosine nitration in cell signaling and cancer. J. Biol. Chem..

[72] Sampson J.B., Ye Y., Rosen H., Beckman J.S. (1998). Myeloperoxidase and horseradish peroxidase catalyze tyrosine nitration in proteins from nitrite and hydrogen peroxide. Arch. Biochem. Biophys..

[73] Abello N., Kerstjens H.A., Postma D.S., Bischoff R. (2009). Protein tyrosine nitration: Selectivity, physicochemical and biological consequences, denitration, and proteomics methods for the identification of tyrosine-nitrated proteins. J. Proteome Res..

[74] Batthyány C., Bartesaghi S., Mastrogiovanni M., Lima A., Demicheli V., Radi R. (2017). Tyrosine-Nitrated Proteins: Proteomic and Bioanalytical Aspects. Antioxid. Redox Signal.

[75] Ohmori H., Oka M., Nishikawa Y., Shigemitsu H., Takeuchi M., Magari M., Kanayama N. (2005). Immunogenicity of autologous IgG bearing the inflammation-associated marker 3-nitrotyrosine. Immunol. Lett..

[76] Thomson L., Christie J., Vadseth C., Lanken P.N., Fu X., Hazen S.L., Ischiropoulos H. (2007). Identification of immunoglobulins that recognize 3-nitrotyrosine in patients with acute lung injury after major trauma. Am. J. Respir. Cell Mol. Biol..

[77] Birnboim H.C., Lemay A.M., Lam D.K., Goldstein R., Webb J.R. (2003). Cutting edge: MHC class II-restricted peptides containing the inflammation-associated marker 3-nitrotyrosine evade central tolerance and elicit a robust cell-mediated immune response. J. Immunol..

[78] Dixit K., Khan M.A., Sharma Y.D., Moinuddin, Alam K. (2011). Peroxynitrite-induced modification of H2A histone presents epitopes which are strongly bound by human anti-DNA autoantibodies: Role of peroxynitrite-modified-H2A in SLE induction and progression. Hum. Immunol..

[79] Ahsan H. (2013). 3-Nitrotyrosine: A biomarker of nitrogen free radical species modified proteins in systemic autoimmunogenic conditions. Hum. Immunol..

[80] Souza J.M., Daikhin E., Yudkoff M., Raman C.S., Ischiropoulos H. (1999). Factors determining the selectivity of protein tyrosine nitration. Arch. Biochem. Biophys..

[81] Bandookwala M., Sengupta P. (2020). 3-Nitrotyrosine: A versatile oxidative stress biomarker for major neurodegenerative diseases. Int. J. Neurosci..

[82] Daiber A., Münzel T. (2012). Increased circulating levels of 3-nitrotyrosine autoantibodies: Marker for or maker of cardiovascular disease?. Circulation.

[83] Teixeira D., Fernandes R., Prudêncio C., Vieira M. (2016). 3-Nitrotyrosine quantification methods: Current concepts and future challenges. Biochimie.

[84] Bandookwala M., Thakkar D., Sengupta P. (2020). Advancements in the Analytical Quantification of Nitroxidative Stress Biomarker 3-Nitrotyrosine in Biological Matrices. Crit. Rev. Anal. Chem..

[85] Hess D.T., Matsumoto A., Kim S.O., Marshall H.E., Stamler J.S. (2005). Protein S-nitrosylation: Purview and parameters. Nat. Rev. Mol. Cell Biol..

[86] Tannenbaum S.R., White F.M. (2006). Regulation and specificity of S-nitrosylation and denitrosylation. ACS Chem. Biol..

[87] Anand P., Stamler J.S. (2012). Enzymatic mechanisms regulating protein S-nitrosylation: Implications in health and disease. J. Mol. Med..

[88] Choi M.S., Jung J.Y., Kim H.J., Ham M.R., Lee T.R., Shin D.W. (2016). S-nitrosylation of fatty acid synthase regulates its activity through dimerization. J. Lipid Res..

[89] Stykel M.G., Ryan S.D. (2024). Network analysis of S-nitrosylated synaptic proteins demonstrates unique roles in health and disease. Biochim. Biophys. Acta Mol. Cell Res..

[90] Nuriel T., Hansler A., Gross S.S. (2011). Protein nitrotryptophan: Formation, significance and identification. J. Proteomics.

[91] Ikeda K., Yukihiro Hiraoka B., Iwai H., Matsumoto T., Mineki R., Taka H., Takamori K., Ogawa H., Yamakura F. (2007). Detection of 6-nitrotryptophan in proteins by Western blot analysis and its application for peroxynitrite-treated PC12 cells. Nitric Oxide.

[92] Bellmaine S., Schnellbaecher A., Zimmer A. (2020). Reactivity and degradation products of tryptophan in solution and proteins. Free Radic. Biol. Med..

[93] Predonzani A., Calì B., Agnellini A.H., Molon B. (2015). Spotlights on immunological effects of reactive nitrogen species: When inflammation says nitric oxide. World J. Exp. Med..

[94] Bregere C., Rebrin I., Sohal R.S. (2008). Detection and characterization of in vivo nitration and oxidation of tryptophan residues in proteins. Methods Enzymol..

[95] Kim G., Weiss S.J., Levine R.L. (2014). Methionine oxidation and reduction in proteins. Biochim. Biophys. Acta.

[96] Drazic A., Winter J. (2014). The physiological role of reversible methionine oxidation. Biochim. Biophys. Acta.

[97] Lourenço Dos Santos S., Petropoulos I., Friguet B. (2018). The Oxidized Protein Repair Enzymes Methionine Sulfoxide Reductases and Their Roles in Protecting against Oxidative Stress, in Ageing and in Regulating Protein Function. Antioxidants.

[98] Fabisiak J.P., Sedlov A., Kagan V.E. (2002). Quantification of oxidative/nitrosative modification of CYS(34) in human serum albumin using a fluorescence-based SDS-PAGE assay. Antioxid. Redox Signal..

[99] Colombo G., Clerici M., Giustarini D., Rossi R., Milzani A., Dalle-Donne I. (2012). Redox albuminomics: Oxidized albumin in human diseases. Antioxid. Redox Signal..

[100] Arfat M.Y., Arif Z., Chaturvedi S.K., Moinuddin, Alam K. (2016). Peroxynitrite-induced structural perturbations in human IgG: A physicochemical study. Arch. Biochem. Biophys..

[101] Iizumi K., Kawasaki H., Shigenaga A., Tominaga M., Otsu A., Kamo A., Kamata Y., Takamori K., Yamakura F. (2018). Tryptophan nitration of immunoglobulin light chain as a new possible biomarker for atopic dermatitis. J. Clin. Biochem. Nutr..

[102] Gole M.D., Souza J.M., Choi I., Hertkorn C., Malcolm S., Foust R.F., Finkel B., Lanken P.N., Ischiropoulos H. (2000). Plasma proteins modified by tyrosine nitration in acute respiratory distress syndrome. Am. J. Physiol. Lung Cell Mol. Physiol..

[103] Pignatelli B., Li C.Q., Boffetta P., Chen Q., Ahrens W., Nyberg F., Mukeria A., Bruske-Hohlfeld I., Fortes C., Constantinescu V. (2001). Nitrated and oxidized plasma proteins in smokers and lung cancer patients. Cancer Res..

[104] Nowak P., Wachowicz B. (2002). Peroxynitrite-mediated modification of fibrinogen affects platelet aggregation and adhesion. Platelets.

[105] Nowak P., Zbikowska H.M., Ponczek M., Kolodziejczyk J., Wachowicz B. (2007). Different vulnerability of fibrinogen subunits to oxidative/nitrative modifications induced by peroxynitrite: Functional consequences. Thromb. Res..

[106] Ponczek M.B., Nowak P., Wachowicz B. (2008). The effects of nitronium ion on nitration, carbonylation and coagulation of human fibrinogen. Gen. Physiol. Biophys..

[107] Bijak M., Nowak P., Borowiecka M., Ponczek M.B., Żbikowska H.M., Wachowicz B. (2012). Protective effects of (-)-epicatechin against nitrative modifications of fibrinogen. Thromb. Res..

[108] Bijak M., Saluk J., Antosik A., Ponczek M.B., Żbikowska H.M., Borowiecka M., Nowak P. (2013). Aronia melanocarpa as a protector against nitration of fibrinogen. Int. J. Biol. Macromol..

[109] Ding Y., Luo Y., Fu J. (2014). Effects of Mn (II) on peroxynitrite nitrifying fibrinogen. Biomed. Mater. Eng..

[110] Ill-Raga G., Palomer E., Ramos-Fernández E., Guix F.X., Bosch-Morató M., Guivernau B., Tajes M., Valls-Comamala V., Jiménez-Conde J., Ois A. (2015). Fibrinogen nitrotyrosination after ischemic stroke impairs thrombolysis and promotes neuronal death. Biochim. Biophys. Acta.

[111] Helms C.C., Kapadia S., Gilmore A.C., Lu Z., Basu S., Kim-Shapiro D.B. (2017). Exposure of fibrinogen and thrombin to nitric oxide donor ProliNONOate affects fibrin clot properties. Blood Coagul. Fibrinolysis.

[112] Marchelak A., Kolodziejczyk-Czepas J., Wasielewska P., Nowak P., Olszewska M.A. (2021). The Effects of *Prunus spinosa* L. Flower Extracts, Model Polyphenols and Phenolic Metabolites on Oxidative/Nitrative Modifications of Human Plasma Components with Particular Emphasis on Fibrinogen In Vitro. Antioxidants.

[113] Rutkowska M., Kolodziejczyk-Czepas J., Olszewska M.A. (2021). The Effects of *Sorbus aucuparia* L. Fruit Extracts on Oxidative/Nitrative Modifications of Human Fibrinogen, Impact on Enzymatic Properties of Thrombin, and Hyaluronidase Activity In Vitro. Antioxidants.

[114] Medeiros R., Sousa B., Rossi S., Afonso C., Bonino L., Pitt A., López E., Spickett C., Borthagaray G. (2021). Identification and relative quantification of 3-nitrotyrosine residues in fibrinogen nitrated in vitro and fibrinogen from ischemic stroke patient plasma using LC-MS/MS. Free Radic. Biol. Med..

[115] Farhana A., Alsrhani A., Khan Y.S., Salahuddin M., Sayeed M.U., Rasheed Z. (2024). Apigenin Provides Structural Protection to Human Fibrinogen against Nitrosative Stress: Biochemical and Molecular Insights. Biomolecules.

[116] Parastatidis I., Thomson L., Burke A., Chernysh I., Nagaswami C., Visser J., Stamer S., Liebler D.C., Koliakos G., Heijnen H.F. (2008). Fibrinogen beta-chain tyrosine nitration is a prothrombotic risk factor. J. Biol. Chem..

[117] Heffron S.P., Parastatidis I., Cuchel M., Wolfe M.L., Tadesse M.G., Mohler E.R., Ischiropoulos H., Rader D.J., Reilly M.P. (2009). Inflammation induces fibrinogen nitration in experimental human endotoxemia. Free Radic. Biol. Med..

[118] Nowak W., Treliński J., Chojnowski K., Matczak J., Robak M., Misiewicz M., Nowak P. (2017). Assessment of oxidative/nitrative modifications of plasma proteins, selected ROTEM parameters and kinetics of fibrinogen polymerization in patients with multiple myeloma at diagnosis. Med. Oncol..

[119] Kanski J., Behring A., Pelling J., Schöneich C. (2005). Proteomic identification of 3-nitrotyrosine-containing rat cardiac proteins: Effects of biological aging. Am. J. Physiol. Heart Circ. Physiol..

[120] Fukuyama N., Takebayashi Y., Hida M., Ishida H., Ichimori K., Nakazawa H. (1997). Clinical evidence of peroxynitrite formation in chronic renal failure patients with septic shock. Free Radic. Biol. Med..

[121] Souza J.M., Peluffo G., Radi R. (2008). Protein tyrosine nitration--functional alteration or just a biomarker?. Free Radic. Biol. Med..

[122] Ischiropoulos H. (2003). Biological selectivity and functional aspects of protein tyrosine nitration. Biochem. Biophys. Res. Commun..

[123] Renner W., Cichocki L., Forjanics A., Köppel H., Gasser R., Pilger E. (2002). G-455A polymorphism of the fibrinogen beta gene and deep vein thrombosis. Eur. J. Clin. Investig..

[124] Hu X., Wang J., Li Y., Wu J., Qiao S., Xu S., Huang J., Chen L. (2017). The β-fibrinogen gene 455G/A polymorphism associated with cardioembolic stroke in atrial fibrillation with low CHA. Sci. Rep..

[125] Brown E.T., Fuller G.M. (1998). Detection of a complex that associates with the Bbeta fibrinogen G-455-A polymorphism. Blood.

[126] Macrae F.L., Domingues M.M., Casini A., Ariëns R.A. (2016). The (Patho)physiology of Fibrinogen γ′. Semin. Thromb. Hemost..

[127] Falls L.A., Farrell D.H. (1997). Resistance of gammaA/gamma′ fibrin clots to fibrinolysis. J. Biol. Chem..

[128] Cooper A.V., Standeven K.F., Ariëns R.A. (2003). Fibrinogen gamma-chain splice variant gamma′ alters fibrin formation and structure. Blood.

[129] Walton B.L., Getz T.M., Bergmeier W., Lin F.C., Uitte de Willige S., Wolberg A.S. (2014). The fibrinogen γA/γ′ isoform does not promote acute arterial thrombosis in mice. J. Thromb. Haemost..

[130] Farrell D.H., Rick E.A., Dewey E.N., Schreiber M.A., Rowell S.E. (2020). γ′ fibrinogen levels are associated with blood clot strength in traumatic brain injury patients. Am. J. Surg..

[131] Allan P., Uitte de Willige S., Abou-Saleh R.H., Connell S.D., Ariëns R.A. (2012). Evidence that fibrinogen γ′ directly interferes with protofibril growth: Implications for fibrin structure and clot stiffness. J. Thromb. Haemost..

[132] Simurda T., Brunclikova M., Asselta R., Caccia S., Zolkova J., Kolkova Z., Loderer D., Skornova I., Hudecek J., Lasabova Z. (2020). Genetic Variants in the FGB and FGG Genes Mapping in the Beta and Gamma Nodules of the Fibrinogen Molecule in Congenital Quantitative Fibrinogen Disorders Associated with a Thrombotic Phenotype. Int. J. Mol. Sci..

[133] Sovova Z., Suttnar J., Dyr J.E. (2021). Molecular Dynamic Simulations Suggest That Metabolite-Induced Post-Translational Modifications Alter the Behavior of the Fibrinogen Coiled-Coil Domain. Metabolites.

[134] Tang Z., Wu H., Du D., Wang J., Wang H., Qian W.J., Bigelow D.J., Pounds J.G., Smith R.D., Lin Y. (2010). Sensitive immunoassays of nitrated fibrinogen in human biofluids. Talanta.

[135] Luo Y., Shi J., Li J. (2015). Peroxynitrite induced fibrinogen site identification. Biomed. Mater. Eng..

[136] Meredith S., Parekh G., Towler J., Schouten J., Davis P., Griffiths H., Spickett C. (2014). Mapping nitro-tyrosine modifications in fibrinogen by mass spectrometry as a biomarker for inflammatory disease. Free Radic. Biol. Med..

[137] Weisel J.W., Litvinov R.I. (2013). Mechanisms of fibrin polymerization and clinical implications. Blood.

[138] Becatti M., Mannucci A., Argento F.R., Gitto S., Vizzutti F., Marra F., Taddei N., Fiorillo C., Laffi G. (2020). Super-Resolution Microscopy Reveals an Altered Fibrin Network in Cirrhosis: The Key Role of Oxidative Stress in Fibrinogen Structural Modifications. Antioxidants.

[139] Cyr A.R., Huckaby L.V., Shiva S.S., Zuckerbraun B.S. (2020). Nitric Oxide and Endothelial Dysfunction. Crit. Care Clin..

[140] Elbatreek M.H., Mucke H., Schmidt H.H.H.W. (2021). NOX Inhibitors: From Bench to Naxibs to Bedside. Handb. Exp. Pharmacol..

[141] Sylvester A.L., Zhang D.X., Ran S., Zinkevich N.S. (2022). Inhibiting NADPH Oxidases to Target Vascular and Other Pathologies: An Update on Recent Experimental and Clinical Studies. Biomolecules.

[142] Li D., Cong Z., Yang C., Zhu X. (2020). Inhibition of LPS-induced Nox2 activation by VAS2870 protects alveolar epithelial cells through eliminating ROS and restoring tight junctions. Biochem. Biophys. Res. Commun..

[143] Forman H.J., Zhang H. (2021). Targeting oxidative stress in disease: Promise and limitations of antioxidant therapy. Nat. Rev. Drug Discov..

[144] Jomova K., Alomar S.Y., Alwasel S.H., Nepovimova E., Kuca K., Valko M. (2024). Several lines of antioxidant defense against oxidative stress: Antioxidant enzymes, nanomaterials with multiple enzyme-mimicking activities, and low-molecular-weight antioxidants. Arch. Toxicol..

[145] Kashyap P., Shikha D., Thakur M., Aneja A. (2022). Functionality of apigenin as a potent antioxidant with emphasis on bioavailability, metabolism, action mechanism and in vitro and in vivo studies: A review. J. Food Biochem..

[146] Han Y., Zhang T., Su J., Zhao Y., Wang C.C., Li X. (2017). Apigenin attenuates oxidative stress and neuronal apoptosis in early brain injury following subarachnoid hemorrhage. J. Clin. Neurosci..

[147] Natsume M., Osakabe N., Yasuda A., Osawa T., Terao J. (2008). Inhibitory Effects of Conjugated Epicatechin Metabolites on Peroxynitrite-mediated Nitrotyrosine Formation. J. Clin. Biochem. Nutr..

[148] Klotz L.O., Sies H. (2003). Defenses against peroxynitrite: Selenocompounds and flavonoids. Toxicol. Lett..

[149] Dietrich-Muszalska A., Kontek B., Olas B., Rabe-Jabłońska J. (2012). Epicatechin inhibits human plasma lipid peroxidation caused by haloperidol in vitro. Neurochem. Res..

[150] Steffen Y., Schewe T., Sies H. (2005). Epicatechin protects endothelial cells against oxidized LDL and maintains NO synthase. Biochem. Biophys. Res. Commun..

[151] Yang B., Dong Y., Wang F., Zhang Y. (2020). Nanoformulations to Enhance the Bioavailability and Physiological Functions of Polyphenols. Molecules.

[152] Wang X., Liu J., Ma Y., Cui X., Chen C., Zhu G., Sun Y., Tong L. (2023). Development of A Nanostructured Lipid Carrier-Based Drug Delivery Strategy for Apigenin: Experimental Design Based on CCD-RSM and Evaluation against NSCLC In Vitro. Molecules.

[153] Niu L., Li Z., Fan W., Zhong X., Peng M., Liu Z. (2022). Nano-Strategies for Enhancing the Bioavailability of Tea Polyphenols: Preparation, Applications, and Challenges. Foods.

